# Effects of natural and artificial selection on survival of columnar cacti seedlings: the role of adaptation to xeric and mesic environments

**DOI:** 10.1002/ece3.1478

**Published:** 2015-03-25

**Authors:** Susana Guillén, Teresa Terrazas, Alejandro Casas

**Affiliations:** 1 Instituto de Biología, Universidad Nacional Autónoma de México, Ciudad UniversitariaDel. Coyoacán, México, D.F., 04510, México; 2 Centro de Investigaciones en Ecosistemas, Universidad Nacional Autónoma de Méxicocampus Morelia. Antigua Carretera a Pátzcuaro 8711, Morelia, Michoacán, 58190, México

**Keywords:** Columnar cacti, domestication, epicotyl, hypocotyl, seedling development, traditional management

## Abstract

*Escontria chiotilla*, *Polaskia chichipe*, and *Stenocereus pruinosus* are species of Mexican columnar cacti that are economically important because of their edible fruits. These species are managed by gathering fruits from the wild, silvicultural management in agroforestry systems, and cultivation in home gardens. Previous studies reported that artificial selection favored individuals that produced larger fruits, which indirectly led to the production of larger seeds and seedlings, with possible effects on survival. We hypothesized that seedlings from managed populations would be larger but more susceptible to xeric conditions than those from wild populations. We evaluated the effects of artificial and natural selection on seedling survival of the three species in wild and managed populations, which were managed with low and high intensity, respectively. We tested seedling performance in gradients of shade (0, 40, and 80%) and humidity (low and high). A GLM of seedling survival showed significant differences among species, shade, and humidity treatments, with each species having environmental requirements associated with their particular adaptations. High humidity decreased seedling survival of all species, and high solar radiation decreased survival of *S. pruinosus* and *P. chichipe*. The effect of management type was significant only in *S. pruinosus*. Significant differences in the initial growth of seedlings among species were detected with ANOVA. In optimal conditions, the hypocotyl and the cotyledons decreased in size and the epicotyl grew, whereas under stress, these structures remained unchanged. The optimum conditions of shade and humidity varied among species and management types. The seedlings of *S. pruinosus* were the largest and the most susceptible, but in all species, seedlings from managed populations were more susceptible to environmental conditions. Thus, artificial selection influenced the susceptibility of these cacti to xeric environments.

## Introduction

Domestication is an evolutionary process through which managed plant or animal populations become adapted to environments created by humans, diverging from the wild populations in morphological, physiological, reproductive, and genetic aspects (Hawkes 1983; Harlan 1992). Domestication occurs primarily as a consequence of artificial selection, which favors the fitness of organisms with features desirable to people. However, in these domesticated environments, natural selection also influences the survival of organisms with specific characteristics that favor survival, reproduction, or both in the particular environmental conditions (Gepts 2004). Thus, artificial and natural selection both contribute to the adaptation of organisms to different human cultural and natural environments, and through time, both types of selection influence the evolutionary divergence between wild and domesticated populations (Gepts 2004; McKey et al. 2012).

In domesticated plants, a suite of specific traits represents evolutionary convergence in response to artificial selection; these traits are called the “domestication syndrome” (Hawkes 1983; Harlan 1992; Evans 1993). However, the changes associated with domestication are also related to adaptive strategies of survival in anthropogenic environments directed by natural selection. These changes can lead to decreased fitness of domesticated organisms in wild environments (Frary and Doĝanlar 2003).

In areas in which domesticates coexist with their wild relatives and domestication is a dynamic ongoing process that adopts diversified evolutionary routes, populations are commonly found with different degrees or states of domestication (Harlan 1992; Gepts 2004; Casas et al. 2007; Lins-Neto et al. 2014). Rural areas within the main centers of origin of domestication and agriculture are ideal sites to analyze how the processes of domestication operate and how variable the evolutionary contexts are in which they occur. One of these sites is in southern Mexico in Mesoamerica, which is one of the main centers for the origin of agriculture in the New World. A site of particular interest is in the Tehuacán Valley in which archeologists recorded some of the oldest remains of agriculture and domestication in Mexico (MacNeish 1967). Currently, in the Tehuacán Valley, more than 300 native plant species receive some form of traditional management (Blancas et al. 2010, 2013). Of these species, the columnar cacti have been outstanding resources since prehistoric times (MacNeish 1967) because of their diversity and abundance and because most of them provide valuable edible fruits (Casas and Barbera 2002).

For the different species of columnar cacti, Casas et al. (1999a, 2007) identified three primary management regimes: (1) gathering the harvesting of fruits and other useful plant parts from wild populations; (2) in situ or silvicultural management in agroforestry systems in which people clear fields for cultivating crop plants, but they leave wild individuals standing with phenotypes favorable to people, particularly those plants with the best phenotypic characteristics, such as large fruit sizes, attractive colors, sweet flavors, lower amounts of spines, and thin peels, among others; and (3) cultivation in home gardens and other crop fields in which cacti are propagated by vegetative propagules or seeds, which involves artificial selection with the choice of the asexual or sexual propagules based on the attributes of the mother plant. Cultivation is uncommon for plants in which vegetative propagation is difficult but is common in species that combine vegetative propagation and fast growth. Morphometric and population genetics studies that compared wild and managed populations of several species showed divergences among these population types that were proportional to the intensity of the management (Casas et al. 2006, 2007; Parra et al. 2010, 2012; Cruse-Sanders et al. 2013).

The degree of differentiation between wild and domesticated populations is associated with the intensity of artificial selection (Casas et al. 2007) but is also affected by the magnitude of barriers to gene flow between the populations. Because studies of the reproductive biology of the cacti have not found significant temporal or spatial barriers to pollen flow (Casas et al. 1999b; Cruz and Casas 2002; Oaxaca-Villa et al. 2006; Ortíz et al. 2010) between wild and domesticated populations, Casas et al. (1999b) hypothesized that divergences were caused by the differential success of seed germination and seedling establishment in wild and domesticated environments. According to this hypothesis, the seeds and seedlings from cultivated plants germinate and establish successfully in domesticated environments but not in the wild.

In studies that analyzed the adaptation of domesticated organisms in wild and agricultural environments, anthropogenic environments were found generally to have markedly different ecological conditions compared with those of wild environments (Pujol et al. 2005). Therefore, the divergence associated with domestication can be hypothesized to occur in the initial steps of the life cycle, particularly in relation to the optimum conditions of humidity. We previously conducted several common garden studies with columnar cacti species with different degrees of domestication (Guillén et al. 2009, 2011, 2013; Rodríguez-Morales et al. 2013). In those studies, we compared the rates of seed germination and seedling survival in xeric and mesic conditions that simulated wild and cultivated environments, respectively. In those studies, we identified some morphophysiological patterns that were associated with artificial selection and identified others associated with natural selection. We concluded that artificial selection that favored larger fruits had indirect consequences on seedling survival, because larger sized fruits had larger seeds that produced more vigorous seedlings that had differential success in mesic (home gardens) and xeric (wild) environments. However, we do not know how artificial selection influences the development and morphology of seedlings, and this information could be the key to understand the different adaptations for the establishment of seedlings to survive in wild and managed environments.

One of the few studies to analyze the functional morphology of seedlings in the context of domestication was conducted by Pujol et al. (2005) with cassava (*Manihot esculenta* Crantz). Notably, these authors found morphological differences between seedlings from domesticated plants and those from their wild relatives, and the differences were associated with adaptations to the environmental conditions in which seedlings became established. The wild relatives had hypogeal germination, which results in seedlings that successfully survive the risks of fire and drought that characterize wild environments. By contrast, the cultivated plants had epigeal germination, which results in seedlings that have higher growth rates adapted to the more stable conditions of humidity and nutrients in agricultural environments. The seeds and seedlings from domesticated plants did not survive in wild environments. Similar scenarios could be occurring in a number of other systems with domesticated species and their wild relatives. For columnar cacti, seedlings may be affected by artificial selection operating in cultivated environments and by natural selection operating in both wild and cultivated environments; both types of selection could determine the different morpho-anatomical adaptations.

Cacti have numerous adaptations to survive in arid and semiarid environments (Nobel 1988). However, little is known about the adaptations for germination and establishment, which are the steps in the life cycle with the highest rates of mortality (Steenbergh and Lowe 1969; Harper 1977; Valiente-Banuet and Ezcurra 1991; Godínez-Álvarez and Valiente-Banuet 1998) and the strongest influence on the development of population structure (Donohue et al. 2010). Moreover, relatively few studies documented the morpho-anatomical and physiological aspects of the seedling stage. Loza-Cornejo and Terrazas (2011) analyzed the morpho-anatomical changes that occurred during ontogeny to examine the correlations with adaptations to establishment and development. These authors found that each species allocated resources to different organs (the seedlings of species from larger seeds allocated more biomass to roots, cotyledons, and stem growth and were more vigorous, whereas the seedlings of species from smaller seeds produced shorter seedlings that grew more slowly but maintained a low rate of transpiration to tolerate short periods of drought). Therefore, the authors concluded that these observations could be explained as adaptive strategies for survival specific to the environments in which they grow.

We previously reported that seeds of individual domesticated plants are generally larger, although more susceptible to water stress during germination; these larger seeds then produce larger seedlings with lower rates of survival in xeric conditions. However, it is unknown (1) at which stage of development the seedlings from domesticated plants are more susceptible than those from wild plants, (2) whether morphological differences exist between wild and managed seedlings that influence susceptibility to water stress and shade, and (3) whether the differences in survival are related to different environmental requirements. To answer these questions, we analyzed the effects of artificial and natural environments on the functional morphology of seedlings and its relation to survival. The hypothesis of our study was that artificial selection for larger fruits indirectly affected seed size and germination patterns and the initial size of seedlings. Therefore, we postulated that (1) seedlings from managed populations would have higher requirements for shade and humidity than seedlings from wild populations; (2) differences in germination and establishment would be found among species depending on their natural history; and (3) differences would be found in susceptibility to hydric stress and in survival. The analysis was conducted with columnar cacti species along a gradient from lower to higher intensity of management. We expected to find greater divergence between wild and managed populations in the more intensely managed species.

## Materials and Methods

### Species and populations studied

**Figure 1:**
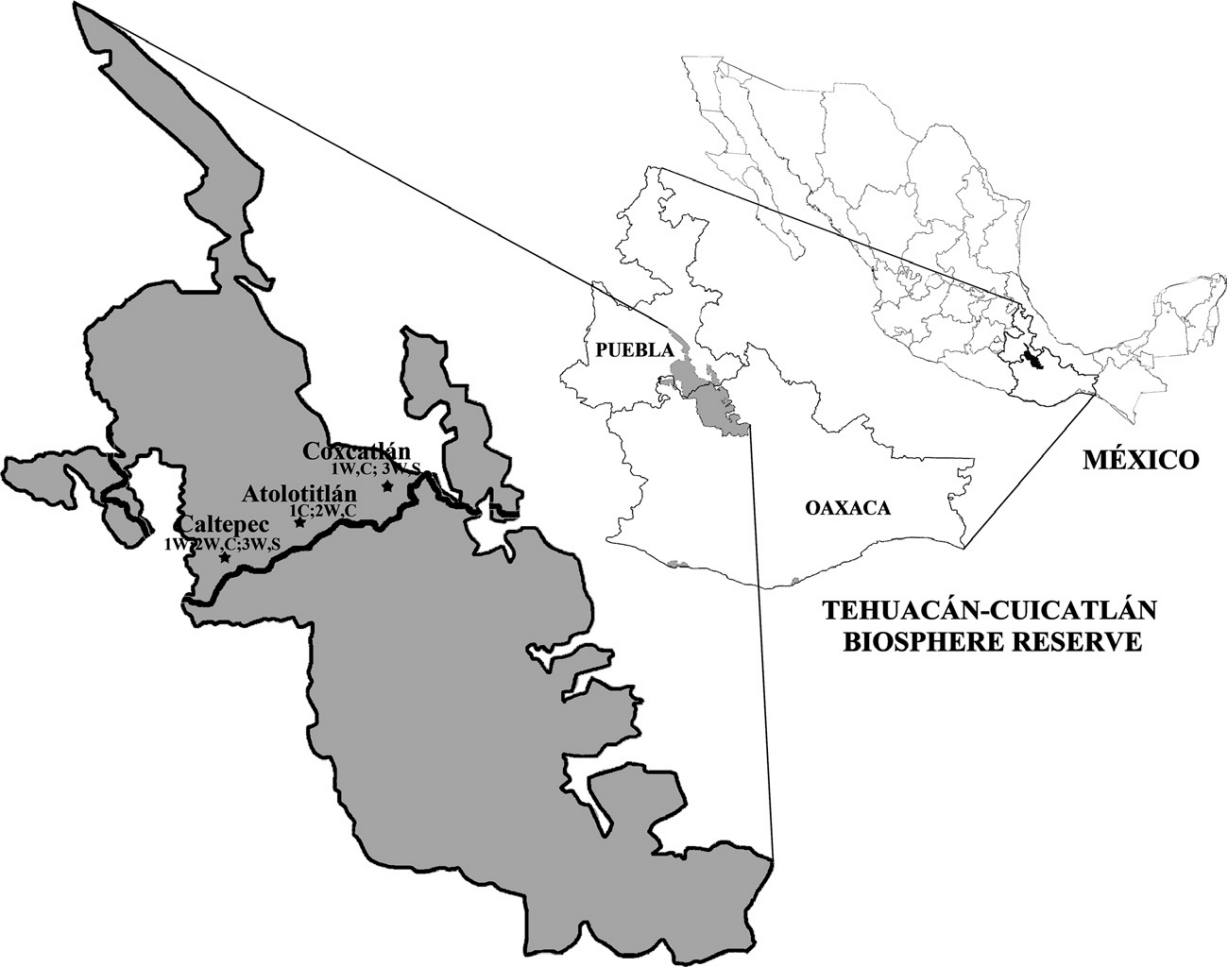
Study area. Location of columnar cacti populations studied in the Tehuacán-Cuicatlán Biosphere Reserve, central México. 1 = *Stenocereus pruinosus*; 2 = *Polaskia chichipe*; and 3 = *Escontria chiotilla*. W = Wild population; S = Silvicultural managed population; and C = Cultivated population.

We studied wild and managed (including both silvicultural and cultivated stands) populations of *Stenocereus pruinosus*, *Polaskia chichipe*, and *Escontria chiotilla* (Cactaceae) in the territory of the villages of San Luis Atolotitlán, Caltepec, and Coxcatlan in the Tehuacán-Cuicatlán Biosphere Reserve, central Mexico (Fig.1). In the region, the climate is semiarid, and in San Luis Atolotitlán and Caltepec, the annual mean temperature is 18°C with rainfall of 655 mm (García 1988), whereas in Coxcatlán, the annual mean temperature and rainfall are 24°C and 441 mm, respectively (Casas et al. 1999b). For each species, we collected seeds from two wild and two cultivated populations, except for *E. chiotilla*, which is not cultivated but silviculturally managed, so we included samples from two managed populations.

*Stenocereus pruinosus* and *E. chiotilla* occur naturally in tropical deciduous forests in alluvial areas in which they are dominant elements alongside other species of columnar cacti, such as *Lemairocereus hollianus* and *Pachycereus weberi* (Casas et al. 1999a). *Polaskia chichipe* is a dominant component of the thorn scrub forest type called “chichipera” described by Valiente-Banuet et al. (2000) and is distributed on soils derived from volcanic rocks.

### Sampling of fruits and seeds

In May 2009, we collected the fruits of *S. pruinosus, P. chichipe,* and *E. chiotilla*. From each population, we collected five to eight fruits from each of 10 randomly chosen individual plants. The seeds from each fruit were stored in paper bags at room temperature.

### Production and management of seedlings

For each species and population type, the seeds were disinfected for 15 min in a 70% solution of Na(ClO)_2_ and were then sown in a soil mixture of similar proportions of sand and peat moss with particles <1 mm. After 60 days from sowing, we measured the height of seedlings with a Mitutoyo Digimatic SR44 digital calliper (MITUTOYO CORPORATION, Kawasaki-shi, Kaganawa, Japan). The seedlings used for the different shade and humidity treatments were planted in pots (10.5 cm diameter, 4 cm depth) with 150 g of substrate (equal portions of sand, peat moss, and volcanic coarse sand with particles <1.5 mm). Each replicate had a total of 66 seedlings.

To prevent deleterious effects of the intense greenhouse climatic conditions on seedling survival, we maintained the seedlings for 30 days in the laboratory and for 15 days in the greenhouse under the same conditions of light and humidity before experimental treatments. Fungal attack was prevented with the application of captan (10 g/L) solution. To identify the differences in vulnerability between wild and managed seedlings after acclimatization, we conducted a second measurement of seedling height. These data, in addition to the data from the first measurement, were used to calculate initial absolute growth rates (AGR _initial_) with the equation shown below.

### Experimental treatments for seedling establishment in xeric and mesic environments

To evaluate the establishment of seedlings of each species in the wild and managed populations, we conducted common garden experiments to evaluate the following factors. (1) Humidity effects were evaluated at two ranges, high humidity 60–80% (HH) and low humidity 40–60% (LH). To determine the treatments, we characterized the real availability of water for plants with a curve of moisture release from the substrate used in the experiment, which was generated by converting the content of water (in percentage) into water potential (MPa). According to this curve, 60–80% humidity corresponded to a water potential of −0.56 to −0.31 MPa, and 40–60% humidity corresponded to −3.0 to −0.56 MPa. The percentage humidity was constant throughout the experiment. We irrigated daily with distilled water for 4 of the 8 months of the study to simulate the natural rainy and dry seasons in the Tehuacán-Cuicatlán Valley. (2) Shade effects were controlled using nets with different openings: none (0%) or complete exposure (CE), 40% (S40%), and 80% (S80%). (3) Management effects were examined on seedlings from wild populations and on seedlings from managed populations (For *E. chiotilla*, cultivated populations and silviculturally managed populations). We established 12 treatments per cactus species combining these factors. Each treatment had three replicates with 66 seedlings each; 34 seedlings were monitored weekly to evaluate survival, and 32 seedlings were monitored monthly to determine the absolute growth rate.

The environmental variables (temperature, radiation, and relative humidity) were continually monitored in each treatment using HOBO H8 sensors (Onset Computer Corporation, Pocasset, MA) placed on the tables used for the different treatments within the greenhouse. Finally, we used a nested design with fully randomized crossed factors (species, management, and humidity) nested in the main factor shade; therefore, all crossed factors were represented in each shade treatment.

### Environmental conditions of the greenhouse

**Figure 2:**
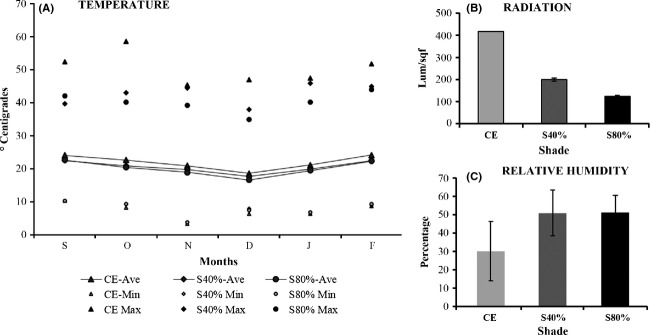
Temperature (A), radiation (B), and relative humidity (C) registered in the greenhouse (where the experiments were conducted) under three percentages of shade (complete exposure = CE; shade 40% = S40%; and shade 80% = S80%) between September 2010 and February 2011.

The experiment was conducted for 8 months. However, we had sufficient replicates to perform the statistical analyses only until the sixth month. During the 6 months of the experiment, the average monthly temperatures were high (Fig.2). The highest average temperature was recorded in September (CE = 24.01°C, S40% = 22.62°C and S80% = 22.42°C). The maximum temperatures recorded decreased significantly under the different shade treatments (CE = 58.55°C, S40% = 42.94°C, and S80% = 40.10°C). The differences in minimum temperatures were not significant among shade treatments (CE = 3.31°C, S40% = 3.74°C, and S80% = 3.81°C). The solar radiation did not vary significantly within each light treatment over the experimental period but did vary significantly among shade treatments (CE = 394.70, S40% = 175.92, and S80% = 132.25 lum/ft^2^). The relative humidity in the experimental environments was lowest in the CE treatment (26.16%) and highest in the S80% treatment (53.78%).

### Measurement of morphological traits

Four seedlings per replicate were sampled to evaluate growth. For each seedling, the lengths of the hypocotyl, epicotyl, cotyledons, and roots were measured following the method used by Ayala-Cordero et al. (2006). The measurements were conducted before seedlings were submitted to treatments and again at the conclusion of the experiment; both measurements were used to calculate the AGRs (absolute growth rate) of the different morphological traits.

### Initial absolute growth rate and growth rates of parts of seedlings

During the experiment, two critical points were identified at which the differences in growth and survival between wild and managed populations were markedly evident. The first critical point occurred before seedlings were established with the experimental treatments (105 das [days after sowing]); at this critical point, the initial absolute growth rate (AGR_initial,_ in mm/day) was calculated using seedling height with the following equation:

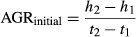
where *h*_1_ and *h*_2_ are the height of the seedling at times *t*_1_ and *t*_*2*_, in days.

The second critical point occurred 5 months (255 das) after the seedlings were established in the treatments, and the absolute growth rates of the different parts of the seedlings (AGR _hypocotyl_, AGR _epicotyl_, AGR _cotyledons_ and AGR _roots_) were calculated with the same equation used for AGR _initial._ Finally, survival was evaluated.

### Statistical analyses

The data for seedling AGR _initial_ were compared with a factorial ANOVA, and the growth of the different traits (AGR _hypocotyl_, AGR _epicotyl_, AGR _cotyledons_, and AGR _roots_) was compared with a multivariate ANOVA (MANOVA). In both cases, all crossed factors were nested in the factor shade to evaluate both inter- and intraspecific differences. To analyze intraspecific differences, we considered the categorical variables shade, management, and humidity, whereas to analyze differences among species, we also included the categorical variable species. The assumptions of ANOVA were evaluated using the Shapiro–Wilk test for normality. The data without homogeneity of variance were square root transformed (Sokal and Rohlf 1995).

The data on seedling survival were analyzed with generalized linear models (GLM) with a binomial link function (McCullagh and Nelder 1989). The most parsimonious model was selected, and the significance of each factor was evaluated with a deviance analysis. To evaluate the association of variation in the growth rates of the different parts of the seedling with survival, we conducted correlation analysis in each treatment. All analyses were performed using the statistical program R (version 2.13 2, R Development Core Team 2008).

## Results

### Absolute growth rate (initial)

**Figure 3:**
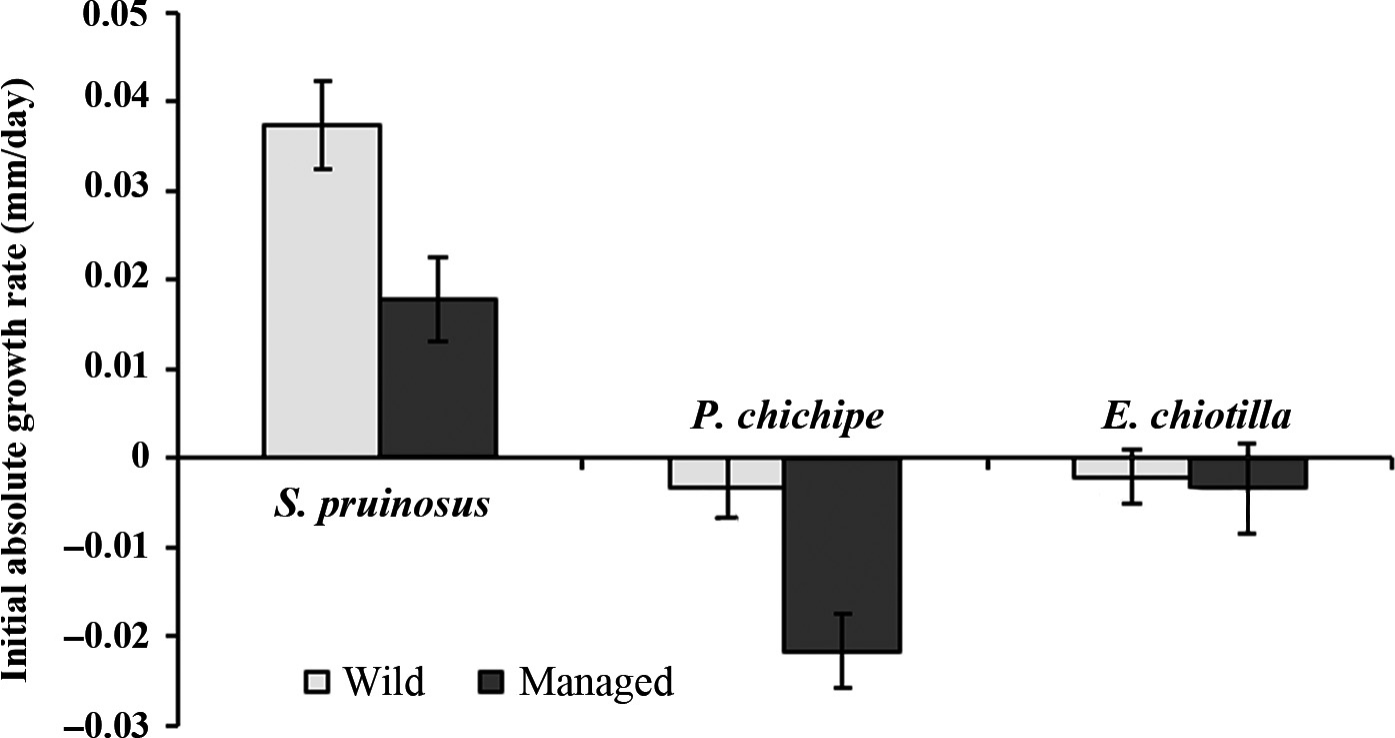
Initial absolute growth rate (105 days from seed planted) ± SE of different columnar cacti from wild and managed populations.

The AGR _initial_ was significantly different among the three species (Table1). The highest AGR _initial_ was for the growth of *S. pruinosus* seedlings, whereas the seedlings of *P. chichipe* and *E. chiotilla* initially decreased in size (Fig.3). For the three species, seedlings from the managed populations had the lowest growth rates, and significant differences for AGR _initial_ were found only among the species that were more intensively managed (Table1).

**Table 1:** ANOVA of interspecific and intraspecific differences for the first critical point (105 das) in initial absolute growth rates (AGR _initial_) of seedlings (*n* = 72) from wild and managed populations

Intraspecific analysis	
Species	*F*_2,142_
*Stenocereus pruinosus*	8.166**
*Polaskia chichipe*	11.786***
*Escontria chiotilla*	0.046 NS

Interspecific analysis	
Factor	*F*_2,426_
*Species*	47.519***
*Management*	13.870***
Species*Management	2.832 NS

NS, Not significant.

* *P *<* *0.05

** *P *<* *0.01

*** *P *<* *0.001.

### AGR of interspecific morphological structures

**Figure 4:**
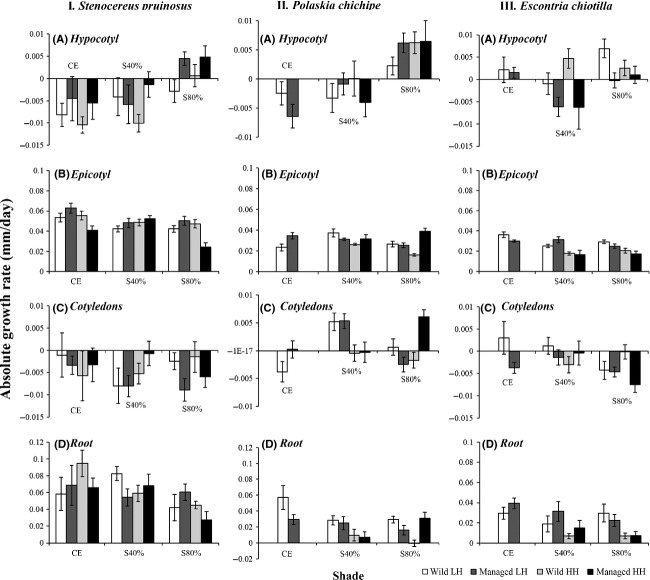
Absolute growth rate ± SE of different morphological structures (hypocotyl, epicotyl, cotyledons, and roots) of seedlings from wild and managed populations of *Stenocereus pruinosus*, *Polaskia chichipe,* and *Escontria chiotilla* after 5 months under different percentages of shade (complete exposure (CE), shade 40% (S40%), and shade 80% (S80%)) and low humidity (LH = 60–40%) and high humidity (HH = 80–60%).

According to the MANOVA, the differences were significant for the absolute growth rates among the different factors of shade, species, and humidity for all the morphological structures (Table2). *Stenocereus pruinosus* had the highest growth rates of all structures compared with *P. chichipe* and *E. chiotilla*, and these two latter species had similar growth rates of their structures (Fig.4).

**Table 2:** Results of interspecific MANOVA performed during the second critical point on AGRs of different morphological structures of seedlings from wild and managed populations of *Stenocereus pruinosus*, *Polaskia chichipe*, and *Escontria chiotilla* after 5 months under different percentages of shade (complete exposure (CE), shade 40% (S40%), and shade 80% (S80%)) and low humidity (LH = 60–40%) and high humidity (HH = 80–60%)

Factor	df	*˜F*	Hypocotyl	Epicotyl	Cotyledons	Root
			*F*	*F*	*F*	*F*
Shade	2	11.357***	20.895***	16.850***	2.378 NS	13.487***
Species	6	11.284***	3.862***	48.505***	5.827***	22.403***
Management	3	1.229 NS	0.709 NS	1.484 NS	2.227 NS	0.505 NS
Humidity	3	2.900***	0.236 NS	7.956***	0.296 NS	5.337**
Species:Management	6	2.463***	3.429**	3.948***	1.136 NS	0.916 NS
Species: Humidity	6	1.918**	0.486 NS	3.752**	2.125 NS	0.983 NS
Shade:Management:Humidity	3	1.096 NS	0.222 NS	1.547 NS	0.907 NS	0.462 NS
Shade:Species:Management:Humidity	5	3.609***	1.827 NS	9.169***	1.399 NS	3.336 NS
Residuals	317					

NS, Not significant.

* *P *<* *0.05

** *P *<* *0.01

*** *P *<* *0.001.

In all species, the hypocotyl and cotyledons decreased in length, whereas the epicotyl grew. In general, the treatments in which the hypocotyl showed the greatest decrease in length were those in which the epicotyl had the highest growth rates, which were also those with higher percentages of survival. The highest rates of root growth were recorded with low humidity and high solar radiation, and *S. pruinosus* had the highest growth rate. The relationship between AGR of cotyledons and survival was different among the three species.

### Stenocereus pruinosus

#### Hypocotyl

The MANOVA showed significant differences in AGRs among the different shade treatments (Table3). In all treatments, we recorded wide variation in the results (Fig.4-IA). The hypocotyls decreased in size; the greatest decrease was in the CE treatment, and the lowest decrease was in the S80% treatment in which hypocotyls from cultivated plants actually increased in size.

**Table 3:** Results of intraspecific MANOVA performed during the second critical point on absolute growth rates (AGR) of different morphological structures of seedlings from wild and managed populations of columnar cacti after 5 mo under different percentages of shade (complete exposure (CE, 0%), shade 40% (S40%), and shade 80% (S80%)) and low humidity (LH = 60–40%) and high humidity (HH = 80–60%)

Factor	df	*˜F*	Hypocotyl	Epicotyl	Cotyledons	Root
			*F*	*F*	*F*	*F*
*Stenocereus pruinosus*						
Shade	2	4.281***	9.116***	6.829**	0.385 NS	6.109**
Shade × Management	3	1.535 NS	2.078 NS	2.453 NS	1.307 NS	0.389 NS
Shade × Humidity	3	1.948*	2.219 NS	4.124**	1.260 NS	1. 294 NS
Shade × Management × Humidity	3	2.789 **	1.123 NS	7.656***	0.351 NS	2.334 NS
Residuals	112					
*Polaskia chichipe*						
Shade	2	8.964***	16.170***	8.198***	5.129**	6.641**
Shade × Management	3	2.767**	0.378 NS	7.247***	2.586 NS	1.542 NS
Shade × Humidity	3	2.478**	0.641 NS	1.993 NS	4.771**	4.129**
Shade × Management × Humidity	3	2.289**	1.503 NS	6.149***	3.098*	3.044 *
Residuals	104					
*Escontria chiotilla*						
Shade	2	5.008***	4.092*	5.911**	3.580*	4.385*
Shade × Management	3	1.774 NS	4.564**	1.436 NS	1.075 NS	1.225 NS
Shade × Humidity	3	2.899***	0.524 NS	10.153***	0.486 NS	3.986**
Shade × Management × Humidity	3	1.998*	1.213 NS	1.156 NS	2.517 NS	0.198 NS
Residuals	101					

NS, Not significant.

* *P *<* *0.05

** *P *<* *0.01

*** *P *<* *0.001.

The humidity affected hypocotyl growth in wild and cultivated plants in different ways (Fig.4-IA). The seedlings of cultivated plants under higher humidity had a lower decrease in hypocotyl size throughout the shade gradient, and in the S80% treatment, the hypocotyl grew more than in wild plants in which the hypocotyl increased in size only in some seedlings. The seedlings of both wild and cultivated plants grown at high humidity had a lower percentage of survival.

#### Epicotyl

The epicotyl grew in all shade treatments, but the growth rate was the highest in the CE treatment (Fig.4-IB). The MANOVA found significant differences in AGRs among shade treatments (Table3). In CE and S80% treatments, the epicotyls of seedlings from cultivated plants with higher humidity had lower AGRs than wild plants. In seedlings from wild plants, in all shade treatments, the epicotyl grew more with higher humidity, whereas in seedlings of cultivated plants, the humidity did not have a significant effect. The MANOVA showed that the interaction Shade × Management × Humidity was significant (Table3).

#### Cotyledons

In all shade treatments, the cotyledons decreased in size, but the extent of the decrease was highly variable (Fig.4-IC), and the MANOVA did not find significant differences among the different treatments (Table3). In the CE treatment, the cotyledons of the cultivated plants decreased in size less than those of wild plants. In the S40% treatment, the survival of seedlings was inversely proportional to the decrease in cotyledon size (Fig.4-IC, Table3).

#### Root

Roots grew in the CE and S40% treatments, but there was no clear pattern associated with intensity of management (Table3).

### Polaskia chichipe

#### Hypocotyl

The hypocotyls decreased in size in the CE and S40% treatments, whereas in the S80% treatment, they grew (Fig.4-IIA). The MANOVA showed significant differences in AGRs among the different shade treatments (Table3). In the CE treatment, only the seedlings grown at low humidity survived. The hypocotyls of seedlings from cultivated plants decreased in size, more than the decrease in wild plants. The seedlings of cultivated plants also had the highest percentages of survival (Fig.4-IIA). The effects of humidity on hypocotyl growth differed between the wild and cultivated plants. In the two shade treatments, higher humidity led to a less marked decrease than lower humidity on hypocotyl length in seedlings from wild plants, and in some cases, the hypocotyl increased in length. In seedlings from cultivated plants, hypocotyl length showed the largest decrease in length in the S40% treatment, whereas in the S80% treatment, the hypocotyl increased in length. In the treatment with the most shade, the seedlings from wild plants had the smallest increase in length of the hypocotyl and also the highest survival rate (Fig.4-IIA).

#### Epicotyl

In all treatments, for seedlings from both wild and cultivated plants, the epicotyl increased in length over the course of the experiment (Fig.4-IIB). In the CE treatment, the growth rate of the epicotyl was greater in seedlings from cultivated plants than in seedlings from wild plants. Humidity and management both affected growth. In the S40% treatment, the epicotyl showed the greatest increase in length in seedlings grown at lower humidity, independent of management. In the S80% treatment, the seedlings from wild plants with higher humidity had the lowest growth rate, whereas seedlings from cultivated plants showed the opposite pattern. The MANOVA detected significant differences in AGRs among the different shade treatments, managements, and the interaction of Management × Humidity (Table3).

#### Cotyledons

Throughout the shade gradient, the seedlings in which the cotyledons grew or remained constant had a higher percentage of survival (Table3, Fig.4-IIC). The seedlings with higher humidity showed a decrease in size of cotyledons, and their survival was also lower. In the S80% treatment, the seedlings from wild plants with higher humidity were more vulnerable than those from cultivated plants. The MANOVA showed significant differences in AGRs among the different treatments of shade and humidity and in the interaction of Management × Humidity (Table3).

#### Root

The roots increased in size in all shade treatments. However, root growth was higher in the xeric environment (Table3, Fig.4-IID). In the S80% treatment, the seedlings from cultivated plants and higher humidity had a higher AGR.

### Escontria chiotilla

#### Hypocotyl

In the CE and S80% treatments, the hypocotyls grew (Fig.4-IIIA). For the shade gradient treatments, the seedlings from managed plants had greater decreases in size than the seedlings from wild plants. The humidity affected the growth of seedlings from wild and managed plants differently. In the S40% treatment, the hypocotyls of the seedlings from wild plants grew, whereas those of the seedlings from managed plants decreased in size. In the S80% treatment, the pattern was the inverse. The MANOVA showed significant differences in hypocotyl size among the different shade treatments and in the interaction of Shade × Management (Table3).

#### Epicotyl

The highest AGRs were recorded in the CE treatment and in the one with the lowest humidity (Fig.4-IIIB). The MANOVA detected significant differences among the different shade and humidity treatments (Table3).

#### Cotyledons

The cotyledons decreased in size in the shade treatments. In the S80% treatment, the seedlings had the greatest decrease in size (Fig.4-IIIC). The decrease in size of cotyledons was correlated positively with survival. The MANOVA demonstrated significant differences in the AGRs of cotyledons among the shade treatments (Table3).

#### Root

The roots grew in all the shade treatments, but the highest AGRs were in the CE treatment with the lowest humidity (Fig.4-IIID). In the shade treatments, the seedlings in the low humidity treatment grew more, and the MANOVA showed significant differences in root growth among the seedlings of the different treatments of shade and humidity (Table3).

### Survival

The interspecific deviance analysis demonstrated significant differences in survival among species and among treatments of shade and humidity (Table4). On the shade gradient, seedlings in the low humidity treatment had the highest percentages of survival, with the highest percentages in the S40% treatment (Table4). The interaction terms of Shade × Species and Shade × Management were significant, which indicated that the shade and provenance of seedlings were affected differently among the species (Tables4 and 5).

**Table 4:** Morphologic traits associated with survivorship of columnar cacti seedlings after 5 mo under different percentages of shade (complete exposure (CE), shade 40% (S40%), and shade 80% (S80%)) and low humidity (LH = 60–40%) and high humidity (HH = 80–60%)

Shade	Humidity	Management	*Stenocereus pruinosus* %Surv.		*Polaskia chichipe* %Surv.		*Escontria chiotilla* %Surv.	
CE	Low	Wild	67	Hypocotyl	8.8	Hypocotyl	4	-
		Managed	33	-	17	Epicotyl	37	Hypocotyl
	High	Wild	45	Cotyledons	0	Cotyledons	0	-
		Managed	15	Epicotyl	0	Epicotyl	0	-
S40%	Low	Wild	90	Epicotyl	58	Cotyledons	28	Root
		Managed	79	Epicotyl	44	Epicotyl	31	*-*
	High	Wild	68	Hypocotyl	21	Cotyledons	12	Cotyledons
		Managed	62	Epicotyl	34	-	7	Cotyledons
S80%	Low	Wild	85	Root	26	Cotyledons	28	Hypocotyl
		Managed	89	Cotyledons	5	Epicotyl	29	Epicotyl
	High	Wild	69	Hypocotyl	4	Cotyledons	6	Cotyledons
		Managed	44	Epicotyl	10	-	1.9	Epicotyl

Dash means not enough seedlings for measurement.

**Table 5:** Results of interspecific deviance analyses performed on survival of seedlings from wild and managed populations of *Stenocereus pruinosus*, *Polaskia chichipe*, and *Escontria chiotilla* after 5 mo under different percentages of shade (complete exposure (CE), shade 40% (S40%), and shade 80% (S80%)) and low humidity (LH = 60–40%) and high humidity (HH = 80–60%)

Factor	df	χ^2^
Shade	2	36.687***
Species	2	175.833***
Management	1	3.120 NS
Humidity	1	36.131***
Shade × Species	4	33.241***
Shade × Management	2	0.148 NS
Species × Management	2	14.245***
Species × Humidity	2	12.498**

NS, Not significant.

* *P *<* *0.05

** *P *<* *0.01

*** *P *<* *0.001.

The intraspecific analysis of survival showed that for *S. pruinosus,* there were significant differences for the main factors of shade and humidity and for the interactions of Shade × Management and Shade × Humidity. For *P. chichipe*, the differences in survival were significant only for shade and humidity, whereas in *E. chiotilla,* only the humidity was significant (Table6). In the three species, the highest percentage of survival was in the S40% treatment and the least was in the CE treatment. In the three species, the high humidity resulted in a decrease in survival (Table4). For *S. pruinosus* only, there were significant differences in survival associated with management. Particularly in the CE treatment, more seedlings from the wild plants survived than seedlings from the cultivated plants (67% and 33%, respectively), and in the S80% treatment, the seedlings from cultivated plants survived more than those from wild plants (89% and 85%, respectively; Table4).

**Table 6:** Results of intraspecific deviance analyses performed on survival of seedlings from wild and managed populations of columnar cacti after 5 mo under differing percentages of shade (complete exposure (CE), shade 40% (S40%), and shade 80% (S80%)) and low humidity (LH = 60–40%) and high humidity (HH = 80–60%)

	df	*Stenocereus pruinosus*	*Polaskia chichipe*	*Escontria chiotilla*
		χ^2^	χ^2^	χ^2^
Shade	2	54.377***	19.339***	1.225 NS
Humidity	1	35.288***	4.874*	9.364**
Shade × Management	3	17.085***	0.436 NS	1.823 NS
Shade × Humidity	2	9.129*	2.236 NS	0.076 NS
Shade × Management × Humidity	3	5.593 NS	3.294 NS	2.198 NS

NS, Not significant.

* *P *<* *0.05

** *P *<* *0.01

*** *P *<* *0.001.

### Correlation between size of seedling parts and survival

The correlations between seedling parts and survival varied among species and treatments (Table4). In the CE treatment for the three species, the highest survival was correlated with the size of hypocotyl. In the S40% treatment with low humidity for the seedlings of *S. pruinosus* and *P. chichipe*, the epicotyl was the seedling part most correlated with survival. In *E. chiotilla* seedlings in treatments with low humidity, the root was the part most correlated with survival, but in treatments with higher humidity, it was the cotyledons. In the S80% treatments, seedlings from cultivated plants of *S. pruinosus* and those from wild plants of *P. chichipe* with low humidity had the highest survival, which had the highest correlation with cotyledons. In *E. chiotilla*, with similar humidity conditions, the most correlated plant part with survival was the epicotyl in seedlings from managed plants and the hypocotyl in seedlings from wild plants (Table4).

## Discussion

In columnar cacti, germination and seedling establishment are the most critical phases of the life cycle and are also the most vulnerable with the highest rates of mortality (Steenbergh and Lowe 1969; Harper 1977; Valiente-Banuet and Ezcurra 1991; Godínez-Álvarez and Valiente-Banuet 1998). The most important factors that induce germination are humidity and light (Rojas-Aréchiga et al. 2001), and these factors are also important during seedling establishment. Several ecological studies documented that recruitment occurs during periods with favorable conditions of rainfall (Jordan and Nobel 1979; Pierson and Turner 1998; Godínez-Álvarez et al. 2005), or in “safe sites” (sensu Harper et al. 1961), such as cavities in rocks (Peters et al. 2008), and also under the canopy of perennial shrubs that serve as “nurse plants” (Turner et al. 1966; Steenbergh and Lowe 1969). Nurse plants provide microsites that facilitate growth and increase seedling survival by providing protection against direct solar radiation, extreme temperatures, and soil moisture evaporation (Flores and Jurado 2003; Brooker et al. 2008; Martínez-Berdeja and Valverde 2008). In addition to environmental conditions, characteristics intrinsic to a species, such as seed size and dormancy, in turn influence morpho-anatomical and physiological characteristics that are crucial for survival (Godínez-Álvarez et al. 2005; Ayala-Cordero et al. 2006). In this study, we analyzed the effects of domestication on intrinsic characteristics of the species, and the effects of environmental conditions are discussed below.

### Initial absolute growth rate

Before the seedlings were submitted to the experimental treatments, the seedlings experienced a stress period, which affected in different ways the initial growth of the seedlings of the three species. The interspecific differences in susceptibility could be explained by differences in seedling size, which was correlated with differences in seed size (Ayala-Cordero et al. 2006). In our previous studies (Guillén et al. 2011, 2013), we found differences in seed size among species, with the seeds of *S. pruinosus* larger than the seeds of *E. chiotilla*, which are larger than the seeds of *P. chichipe*. Loza-Cornejo and Terrazas (2011) found positive correlations among seed size, seedling size, and seedling vigor. In the present study, we found that the seed size also influenced the degree of susceptibility during a stress event. We observed that the seedlings of *S. pruinosus*, the species with the largest seeds, were least vulnerable during the first critical event and conserved their vigor even after being submitted into the shade and humidity gradient, as we discuss later. These results are similar to those reported by Ayala-Cordero et al. (2006) for *Stenocereus beneckei*. In this species, larger seeds produced more vigorous seedlings that had higher survival rates than those from smaller seeds. However, in our studies, the intraspecific analysis was relevant and showed that seedlings from managed plants, despite their larger size, were generally more susceptible to stress than those from wild plants (Guillén et al. 2013). Moreover, in *E. chiotilla*, the species with the lowest intensity of management, the difference in seedling susceptibility between wild and managed plants was not significant. This result could be explained because management intensity was low and because the divergence in susceptibility and other aspects (morphological, genetic, and germination patterns, according to Casas et al. 2007; Guillén et al. 2011) between wild and managed populations was proportionally low. The greater susceptibility of seedlings from managed plants could be an indicator that these plants lost the capacity of seedlings to face stressful events, despite the fact that they were larger than those from wild plants. Rosas et al. (2012) documented morphological changes in response to water stress in seedlings of *P. chichipe* and *Echinocactus platyacanthus*. The changes recorded were related quantitatively with anatomical traits of the vascular system. These species differed in their strategies to water stress; thus, these species have different developmental reaction norms, and such differences could result from mechanisms of adaptation (Nijhout 2003). In the present study, the greater susceptibility of seedlings from managed plants in xeric environments, despite being larger and more vigorous than those of wild plants, could be caused by anatomical differences between wild and managed seedlings as a result of divergent adaptation to wild and anthropogenic environments, respectively.

### Interspecific differences in growth

After seedlings were submitted to water stress (second critical point), a pattern of development was observed in the three species in which the hypocotyl and cotyledons decreased in size and the epicotyl and roots grew. From the first morphological study performed by Ganong (1898), who referred to the pattern as “ontogeny metamorphosis,” this pattern of development was observed in several cacti species. The same pattern was also been observed in several species of the tribe Pachycereeae (Loza-Cornejo et al. 2003; Loza-Cornejo and Terrazas 2011), suggesting a common pattern in Cactaceae. Furthermore, Loza-Cornejo and Terrazas (2011) suggested that the time at which the cotyledons fuse with the epicotyl varies among species. This trait, in addition to the presence of ribs, could be an indicator of the beginning of the juvenile and the end of the seedling stage. Thus, species differ in their rates of development. In the present study, *S. pruinosus* was the species with the highest rate of development. The differences in rates of development found in the three species could explain the differences in management intensity found for these species in previous studies, in addition to artificial selection (Casas et al. 2006, 2007; Parra et al. 2010, 2012; Cruse-Sanders et al. 2013).

In all species, roots showed the greatest development in xeric conditions. Such a response could be caused by the greater need of plants to take up external humidity in conditions of water stress. However, the interspecific differences found in absolute growth rates of roots can be explained by differences in seed size. Jurado and Westoby (1992), for example, documented that plants of arid environments have larger seeds that allocate a higher proportion of energy to root growth than do the seeds of plants from other wetter environments. This observation could explain the high absolute growth rates observed in seedlings of *S. pruinosus*, the species with the largest seeds.

### Intraspecific patterns in seedling growth

In *S. pruinosus* and *P. chichipe*, there was an inverse relationship between the decrease in size of the hypocotyl and the growth of the epicotyl, and this pattern was correlated with a higher percentage of survival. Seedling growth depends on the shade and humidity conditions, and if these conditions are not optimal, the growth of the seedling stops. In these cases, the cotyledons and hypocotyl can grow or remain unchanged, whereas the epicotyl also stops growth. Such a growth pattern could be explained because the hypocotyl is an energy reservoir, as suggested by Barthlott and Voit (1979), and our data suggest that cotyledons may contribute to photosynthesis, in addition to being an energy reservoir. However, with optimal conditions, cotyledons are a source of alternative energy that is used during seedling growth. However, *E. chiotilla* might have a different survival strategy. Most likely because of its low rate of development, the hypocotyl is the morphological trait that provides energy and structural support.

Because metabolic pathways are plastic responses, Hernández-González and Briones (2007) noted the expression of the CAM or C_3_ metabolic pathways in cacti seedlings depended on environmental conditions. In water or light-stressed conditions, the seedlings remain alive using the C_3_ metabolic pathway, but they change to CAM in optimal conditions because of higher photosynthetic efficiency. This response could explain the quiescent state of seedlings, which remain alive under water or light stress. Thus, the seedlings, particularly those of *E. chiotilla*, remained alive with the C_3_ metabolic pathway and the energy stored in the hypocotyl and cotyledons, waiting for better environmental conditions to continue growth.

The shade and humidity requirements are different for wild and cultivated seedlings; cultivated seedlings of *S. pruinosus* and *P. chichipe* developed better in mesic conditions than wild seedlings. This response could be related with adaptations to the ecological conditions of anthropogenic environments.

### Survival

With optimal environmental conditions, the seedlings develop and grow into the juvenile stage. In all the species, high humidity affected seedling development because of succulent tissue hypertrophy. In our previous studies (Guillén et al. 2009, 2011), we found low rates of germination under high water potentials of −0.2 MPa for *S. pruinosus* and −0.6 MPa for *P. chichipe* and *E. chiotilla*. In this study, the treatment with the highest water availability had a water potential of −0.31 to −0.56 MPa. According to these results, humidity was a critical factor in germination; however, in more advanced stages of seedling development, shade was the most important factor for establishment. Therefore, the requirements for both stages might be discordant (Schupp 1995). Most likely, in the three species, the magnitude of the discordance was related to the particular natural history of each species and also to the intensity of management. This assumption offers an explanation for the differential patterns of recruitment of wild and managed seedlings in wild and domesticated environments (Pérez-Vega 2004).

For *S. pruinosus* and *P. chichipe*, the highest percentages of survival were in the S40% treatment, and for *E. chiotilla*, the effect of shade was not significant. These results could be attributed to the environmental conditions in which the species are naturally distributed. For example, *E. chiotilla* is naturally distributed on pronounced slopes on which nurse plants that can provide protection against solar radiation are scarce. The effect of management on seedling survival was clearly observed in *S. pruinosus*, the species under the highest intensity of management. In xeric conditions, seedlings from cultivated *S. pruinosus* plants showed higher susceptibility than wild plants of this species, whereas in mesic conditions, the opposite pattern was observed. A similar pattern was observed in germination (Guillén et al. 2011) and in a previous evaluation of seedling survival of this species (Guillén et al. 2013).

The higher susceptibility of domesticated seeds and seedlings, despite the larger size, could be explained by a loss of phenotypic plasticity. In seedlings of the wild relatives of *Manihot esculenta*, the germination type and the development of seedlings depend on the depth of seeds in the ground, a plasticity that was lost during the domestication. This observation explains the exclusivity of epigeal germination in domesticated seeds, even when seeds are in total darkness (Pujol et al. 2005; McKey et al. 2012). *Stenocereus pruinosus* is naturally distributed in alluvial soils in the bottom areas of gullies, so this species is adapted to high humidity conditions. However, management also affected the differences in susceptibility between wild and cultivated populations. In this species, the clear adaptations influenced by natural selection and the adaptations influenced by artificial selection (larger seeds and seedlings and more drought susceptibility, respectively) can be distinguished.

### Correlation between morphological traits and survival

The morphological trait related to survival depended on the environmental conditions experienced by the seedlings. In xeric conditions, survival depended on the hypocotyl, which could be explained because this trait is the most important energy reservoir during development (Barthlott and Voit 1979). In optimal conditions of shade and humidity, the epicotyl was important for survival. This result was reasonable because in optimal conditions, seedlings grow and their epicotyl develops into the main stem of the plant in the juvenile stage. When humidity was not the limiting factor, but solar radiation was, survival was explained by cotyledons. This result could be explained because the cotyledons are photosynthetic and survival depends on the products of photosynthesis, which could be associated with changes in the CAM/C_3_ metabolic pathways that were previously discussed.

## Conclusions

The direct selection of larger fruits indirectly affected seed size, germination patterns and seedling size. Moreover, these characteristics were also associated with shade and humidity requirements during seedling development, and finally, all these traits influenced survival. Although each species has its own requirements associated with its natural history, the requirements associated with artificial selection are more evident in those species with higher intensity of management. The differences found in this study explain the morphophysiological and genetic divergences found between wild and managed populations that were documented in previous studies.
